# Myofibrillar protein synthesis rates are increased in chronically exercised skeletal muscle despite decreased anabolic signaling

**DOI:** 10.1038/s41598-022-11621-x

**Published:** 2022-05-09

**Authors:** Henning T. Langer, Daniel West, Joan Senden, Simone Spuler, Luc J. C. van Loon, Keith Baar

**Affiliations:** 1 Charité, Universitätsmedizin Berlin, Berlin, Germany; 2 Department of Physiology and Membrane Biology, University of California, One Shields Avenue, Davis, CA 95616 USA; 3 KITE Research Institute, University Health Network, Toronto, Canada; 4 Faculty of Kinesiology and Physical Education, University of Toronto, Toronto, Canada; 5 Department of Human Biology, NUTRIM School of Nutrition and Translational Research in Metabolism, Maastricht University Medical Centre+, Maastricht, The Netherlands; 6 Neurobiology, Physiology and Behavior, University of California, Davis, CA USA; 7 VA Northern California Health Care System, Mather, CA USA

**Keywords:** Physiology, Proteins

## Abstract

The molecular responses to acute resistance exercise are well characterized. However, how cellular signals change over time to modulate chronic adaptations to more prolonged exercise training is less well understood. We investigated anabolic signaling and muscle protein synthesis rates at several time points after acute and chronic eccentric loading. Adult rat tibialis anterior muscle was stimulated for six sets of ten repetitions, and the muscle was collected at 0 h, 6 h, 18 h and 48 h. In the last group of animals, 48 h after the first exercise bout a second bout was conducted, and the muscle was collected 6 h later (54 h total). In a second experiment, rats were exposed to four exercise sessions over the course of 2 weeks. Anabolic signaling increased robustly 6 h after the first bout returning to baseline between 18 and 48 h. Interestingly, 6 h after the second bout mTORC1 activity was significantly lower than following the first bout. In the chronically exercised rats, we found baseline anabolic signaling was decreased, whereas myofibrillar protein synthesis (MPS) was substantially increased, 48 h after the last bout of exercise. The increase in MPS occurred in the absence of changes to muscle fiber size or mass. In conclusion, we find that anabolic signaling is already diminished after the second bout of acute resistance type exercise. Further, chronic exposure to resistance type exercise training results in decreased basal anabolic signaling but increased overall MPS rates.

## Introduction

Skeletal muscle is a highly plastic tissue whose mass increases in response to repeated exposure to stimuli such as resistance type exercise and nutrition, or decreases in response to stimuli such as unloading, nerve damage, or aging. As a post-mitotic tissue the primary mode by which skeletal muscle mass is thought to increase in adult animals, including humans, is hypertrophy^1,2^. The molecular signals underlying the anabolic response to an acute bout of resistance type exercise are well characterized and correlate with changes in muscle size after chronic training: muscle contractions cause increased activation of the canonical mTORC1 axis, resulting in phosphorylation of S6K1 (Thr389), its downstream target ribosomal protein S6 (Ser240/244) and eventually translation initiation of contractile proteins^3^. As such, molecular signaling through S6K1 is associated with a robust increase in global and myofibrillar protein synthesis after acute exercise^4^. However, how these changes in signaling and muscle protein synthesis govern long term adaptions to exercise and orchestrate increases in muscle size is less clear. Indeed, chronic exposure to resistance type exercise in rats causes a decrease in anabolic signaling through mTORC1 which can be recovered through detraining^5^. Conversely, data in the context of sarcopenia suggests that age-related muscle loss is associated with an overactivation of mTORC1 that, in rodents, can be ameliorated through rapamycin treatment^6–9^.

Adding to the notion that the downregulation or a more transient activation of mTORC1 might be a physiological response to chronic training, gene expression data from humans showed a strong correlation between markers of mTORC1 inhibition and gains in lean mass following chronic resistance type exercise^10^. Furthermore, myofibrillar protein synthesis rates after an acute bout do not consistently predict long-term changes in muscle mass in response to resistance type exercise^11,12^. However, if contractile protein synthesis is measured at a later time point, when exercise-induced muscle damage is less pronounced, myofibrillar protein synthesis rates start to correlate better with the observed long-term changes in muscle mass^13^. This indicates muscle protein synthesis rates reflect an adaptive response to exercise that includes muscle remodeling rather than muscle growth per se.

The goal of this study was to examine the relationship between acute signaling and protein synthesis responses after eccentric contractions and chronic changes in molecular signaling and myofibrillar protein synthesis after repeated exposure. We hypothesized that mTORC1 signaling would progressively decline over the course of the training regime and become inversely related to myofibrillar protein synthesis.

## Methods

### Animals and stimulation

We conducted two sets of experiments. The first consisted of the stimulation of 5 groups of 10 months old, male Fischer 344-Brown Norway rats (each *n* = 5–6) through ten sets of 6 eccentric contractions of the tibialis anterior (TA) at 4–8 V and 100 Hz as described previously^3^. The TA was collected immediately before stimulation or at 6 h, 18 h and 48 h after stimulation. A subset of these samples has been analyzed and published previously^14^. In the fifth group, the animals were stimulated a second time at 48 h and collected 6 h later (i.e., at 54 h). In the second experiment, we stimulated 7-month-old, male Sprague–Dawley (n = 4) with the same parameters outlined above four times over the course of two weeks.To avoid any potential interference of the last stimulation bout with our subsequent analysis of muscle mass (e.g. exercise-induced edema), the TA was collected 48 h after the last bout when the animals were in a fasted state. To facilitate the repeated stimulations, an electrode wire was implanted onto the sciatic nerve above its point of trifurcation running to the back of the neck of the rats^3^. All stimulations and muscle collections were conducted under isoflurane anesthesia (2–3%). Animals were sacrificed by cervical dislocation (first experiment) or cardiac puncture (second experiment). Animals on the second experiment were fed a diet of 20 g chow (ssniff Spezialdiäten GmbH, Soest, Germany) equivalent to 79 kcal day^−1^ to slow weight gain in rats on an ad libitum diet. Experiments were approved and carried out according to the guidelines of the University of California Davis Animal Care and Use Committee (IACUC) (USA) and the Landesamt für Gesundheit und Soziales (LaGeSo) in Berlin (Germany). All animal experiments were in compliance with the ARRIVE guidelines.

### Western blot analysis

Aliquots of frozen powdered tibialis anterior muscles were homogenized in 200 µL sucrose lysis buffer (SLB; 50 mM Tris pH 7.5, 250 mM sucrose, 1 mM EDTA, 1 mM EGTA, 1% Triton X-100, 1% protease inhibitor complex) on a vortexer for 60 min at 4 °C. Following centrifugation at 10,000*g* for 10 min, the supernatant was collected. Protein concentrations were determined in triplicates using the DC protein assay (Bio-Rad, Hercules, CA, USA). Sample concentrations were adjusted using SLB. Following dilution in Laemmli sample buffer, 1 µg protein/µL were denatured at 100 °C for 5 min. Protein (10–15 µg protein per lane) was loaded on 4–20% Criterion TGX Stain-free gels (Bio-Rad), run for 45 min at 200 V and visualized after a UV-induced 1-min reaction to produce fluorescence. Following quantification, proteins were transferred to nitrocellulose or polyvinylidene difluoride (PVDF) membrane at 100 V for 30–60 min, depending on the size of the protein of interest. Efficient transfer was confirmed using Ponceau staining of the membrane. Membranes were then air dried and directly incubated with the primary antibody or washed and blocked in 1% fish skin gelatin dissolved in Tris-buffered saline with 0.1% Tween-20 (TBST) for 1 h. To allow for the probing of multiple antibodies, the membrane was cut above and below the appropriate molecular weight of the protein of interest prior to incubation with the antibody overnight at 4 °C. The next day, membranes were washed and incubated with HRP-conjugated secondary antibodies at 1:5000 (goat) to 1:10,000 (mouse, rabbit) in 1% skim milk-TBST for 1 h at room temperature. Immobilon Western Chemiluminescent HRP substrate (Millipore, Hayward, CA, USA) was then applied to the membranes for protein visualization by chemiluminescence. Image acquisition and band quantification was performed using the ChemiDoc MP System and Image Lab 5.0 software (Bio-Rad). Protein levels of each sample were calculated as band intensities relative to total protein as described previously^15^. Representative pictures of the blots were cropped to display proteins and samples of interest. Unaltered pictures of every membrane and gel can be found in the supplement (Supplementary Information). The following antibodies were used in this study at a concentration of 1 to 1000. Cell Signaling (Cell Signaling Technology, Danvers, MA): Akt (#9272), phospho-S6 Kinase 1 (S6K1) (Thr389) (#9205; lot 16), phospho-ribosomal protein S6 (S6) (Ser240/244) (#5364), phospho-eEF2 (Thr56) (#2331), phospho-4E-binding protein 1 (4E-BP1)(Thr37/46) (#2855); Santa Cruz (Santa Cruz Biotechnology Inc, Dallas, TX): muscle LIM protein/cysteine and glycine-rich protein 3 (mLIM) (#166930; lot E2814); Millipore Sigma (Merck Group): insulin receptor substrate 1 (IRS1) (#06-248; lot 2465193), puromycin (MABE343).

### SUnSET measurement of protein synthesis

Global muscle protein synthesis after acute exercise was assessed using the SUrface SEnsing of Translation (SUnSET) method as described previously^16^. Puromycin was dissolved in sterile saline (0.9% NaCl) and delivered via i.p. injection (0.02 μmol puromycin × g^–1^ body weight) 30 min prior to muscle collection. Puromycin-truncated peptides, reflecting the rate of global muscle protein synthesis, were analyzed by western blot as described above.

### Histological analysis

The blocked TA was cut into sections using a Leica CM3050 S cryostat (Leica Microsystems, Germany) and Gomori trichrome stainings were performed as described previously^17^. Images were collected using the Leica DMI6000 and digital images were processed using the Leica LAS AF software.

### Deuterium oxide labeling

We used a labeling protocol suitable to detect deuterium (^2^H) enrichments in alanine of the myofibrillar protein fraction of skeletal muscle via GC–MS similar to what has been published previously^18,19^. Briefly, 2 weeks after surgery the animals received an intraperitoneal injection of 0.014 mL g^−1^ bodyweight of D2O (99.8% + Atom D, Euriso-Top GmbH Saarbrücken) and 0.9% NaCl. This injection primed the animals and enriched their body water levels to approximately 2.5% D2O. To maintain the label concentration, the rats received drinking water with 4% D2O enrichment for 14 days prior to collection.

### Myofibrillar protein extraction

Myofibrillar protein isolation was performed as described previously^20^. Briefly, 80–120 mg muscle sample of rat TA (n = 4) was weighed into an Eppendorf tube and stored on ice. A standard buffer solution was added to each sample at 10 μL mg^−1^ and the muscle tissue was thoroughly homogenized. Scissors were used to mince the tissue before subsequent homogenization by plastic pestles. To fractionate a pellet rich in myofibrillar- and other structural proteins, the sample was spun at 700*g* for 10 min at 4 °C. The remaining pellet was washed twice with buffer and dH2O, the supernatant was discarded and 1 mL 0.3 NaOH was added to the pellet to further solubilize the myofibrillar proteins and isolate them from collagen. The samples were heated at 50 °C for 30 min. Subsequently the sample was spun at 10,000*g* for 5 min at 4 °C and the supernatant containing the myofibrillar protein was transferred into 4 mL screw-cap glass vials. One milliliter of 1 M PCA was added to each glass vial to precipitate the myofibrillar proteins. After centrifugation, the supernatant was removed, and the pellet washed twice with 500 μL 70% EtOH. After removal of the EtOH, 1.5 mL of 6 M HCL was added to hydrolyze the samples over night at 110 °C. The next day the samples were put in a heating block (120 °C) and dried under a nitrogen steam. To further purify the amino acids, the samples were passed through Dowex exchange resin (AG 50 W-X8 Resin, Bio-Rad) prior to derivatization. After purification, the glass vials were carefully vortexed and put under a nitrogen steam to dry before derivatization. Samples containing the free amino acids of the myofibrillar protein fraction were then converted to their *tert*-butyldimethylsilyl (TBDMS) derivatives via the addition of 50 μL of N-tert-Butyldimethylsilyl-N-methyltrifluoroacetamide (MTBSTFA) and 50 μL of acetonitrile to the sample. Each sample was then incubated for 1 h at 70 °C. The sample was then transferred to 2 mL screwcap chromacol vials (Thermo Fisher Scientific, Schwerte, Germany) suitable for GC–MS injection.

### Plasma protein extraction

To precipitate plasma protein, 40 μL perchloric acid (20%) were added to 360 μL plasma sample. After vortexing, free amino acids were separated from protein bound amino acids by centrifugation (3500 rpm, 20 min, 4 °C). The pellet was collected and washed three times with 1 mL perchloric acid (2%) before being hydrolyzed over night as described above. After hydrolysis, samples were purified and processed for GC–MS injection as described above. Values of unlabeled samples were used as a baseline control for ^2^H enrichment in plasma protein bound alanine.

### Free alanine enrichments in plasma

Plasma samples were thawed on ice and dry 5-sulfosalicylic acid was added to the sample to deproteinize it as described previously^21^. After vortexing, the sample was spun at 1000*g* for 15 min. The supernatant was collected and then purified, processed, and measured on the GC–MS as described in the sections above.

### Gas chromatography–mass spectrometry

The alanine enrichment was determined by electron ionization gas chromatography-mass spectrometry (GC–MS; Agilent 6890N GC/5973N MSD) using selected ion monitoring of masses 232, 233, 234, 235, and 236 for their unlabeled and labeled ^2^H-alanine. We applied standard regression curves to assess linearity of the mass spectrometer and to control for the loss of tracer.

### Calculations

Myofibrillar protein synthesis rates were calculated using the precursor-product method^22,23^.

$${\text{FSR}} \left( {\% *{\text{d}}^{{ - {1}}} } \right) \, = \, (\Delta {\text{MPEmyo}}/(\Delta {\text{MPEplasma}}*{\text{t}})) \, *{1}00$$

where FSR is the fractional synthesis rate of myofibrillar proteins, ΔMPE_myo_ is the change in enrichment of ^2^H in muscle protein-bound alanine, ΔMPE_plasma_ is the change in enrichment of ^2^H in free alanine found in plasma and t is time.

### Statistics

Depending on the number of the groups compared, an unpaired t-test or a two-way analysis of variance (ANOVA) with a post hoc Tukey's multiple comparisons test was used to test the null hypothesis. An alpha of P < 0.05 was deemed statistically significant, and a P value between 0.05 and 0.1 was deemed a trend. Data in the text are reported as mean ± standard deviation, and data in the figures are visually represented as mean ± standard deviation of the mean, scatter dot plot with error bars indicating standard deviation, or box with whiskers where the whiskers indicate the minimal to maximal values, the line indicates the median and the box the 25th to 75th percentile of all values. All analysis was performed with GraphPad Prism Version 8 (La Jolla, CA, USA). All data from our experiments is available in the figures.

## Results

### The effect of a repeated bout of eccentric contractions on anabolic signaling

**Figure 1:**
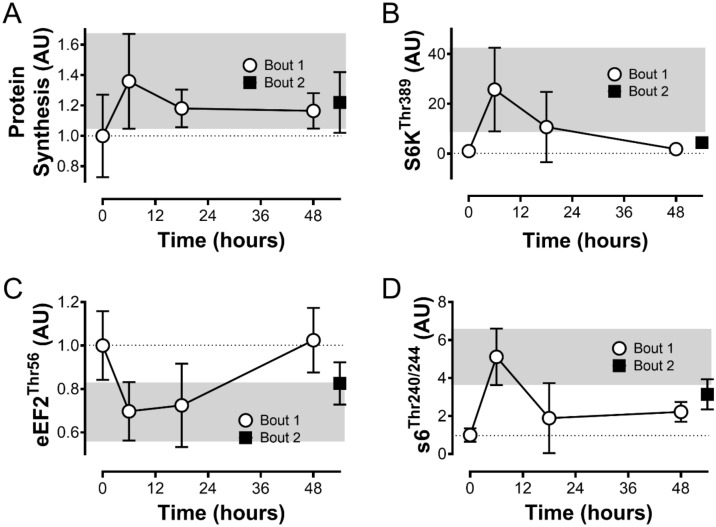
Anabolic signaling in response to an acute exercise bout of eccentric contractions. Protein levels of Puromycin (**A**), phosphorylated S6K1 (Thr389) (**B**), phosphorylated eEF2 (Thr56) (**C**) and phosphorylated S6 (Ser240/244) (**D**). Protein levels are displayed relative to the non-exercised contralateral control leg. Collection time points were at 0 h (no exercise) and after one bout of eccentric contractions at 6 h, 18 h and 48 h (n = 5–6). At 48 h after the first stimulation a second bout with identical parameters was conducted and the animals were collected 6 h later (54 h) (n = 5). Intensity of the bands was normalized to total protein content of the gel or membrane. Data points between 0-48 h have been part of a previous publication by our laboratory^14^. Data is represented as mean ± SD. * indicates a p-value of < 0.05, ** indicates a p-value of < 0.01, and **** indicates a p-value of < 0.0001.

Signaling through the mTORC1 axis was activated significantly following the first acute bout of eccentric contractions. Phosphorylated S6K1 (Thr389) levels increased 26-fold at 6 h (p < 0.01) and decreased back to 11-fold and less than twofold at 18 h and 48 h, respectively (Fig. 1B). The second bout of eccentric contractions at 48 h did not significantly increase p-S6K1 levels 6 h later, at 54 h total time (p > 0.99). Similarly, phosphorylation of S6K1’s downstream target S6 (Ser240/244) increased fivefold at 6 h following eccentric contractions (p < 0.0001), declined back to twofold at 18 h and stayed at about twofold higher at 48 h (Fig. 1D). The second bout did not increase p-S6 levels 6 h after the second bout, at 54 h total time (p = 0.94). At 6 h after eccentric contractions, phosphorylation of translation elongation factor (eEF)2 (Thr56) decreased to about 70% of the control value (p < 0.05) (Fig. 1C). Phosphorylation stayed depressed (73% at 18 h) rising back to baseline at 48 h. Phosphorylation of eEF2 decreased about 18% 6 h after the second bout without reaching statistical significance (p = 0.55). Finally, protein synthesis as assessed via puromycin increased by about 36% at 6 h after the first bout without reaching significance (p = 0.16) (Fig. 1A). Protein levels decreased back to about 18% above baseline at 18 h and stayed around this level at 48 h. The second bout at 48 h did not significantly increase global protein synthesis at 54 h, with puromycin levels staying 21% above baseline levels compared to 0 h. Data points between 0-48 h have been part of a previous publication by our laboratory^14^.

### Chronic exposure to eccentric contractions reduces basal mTORC1 signaling

**Figure 2:**
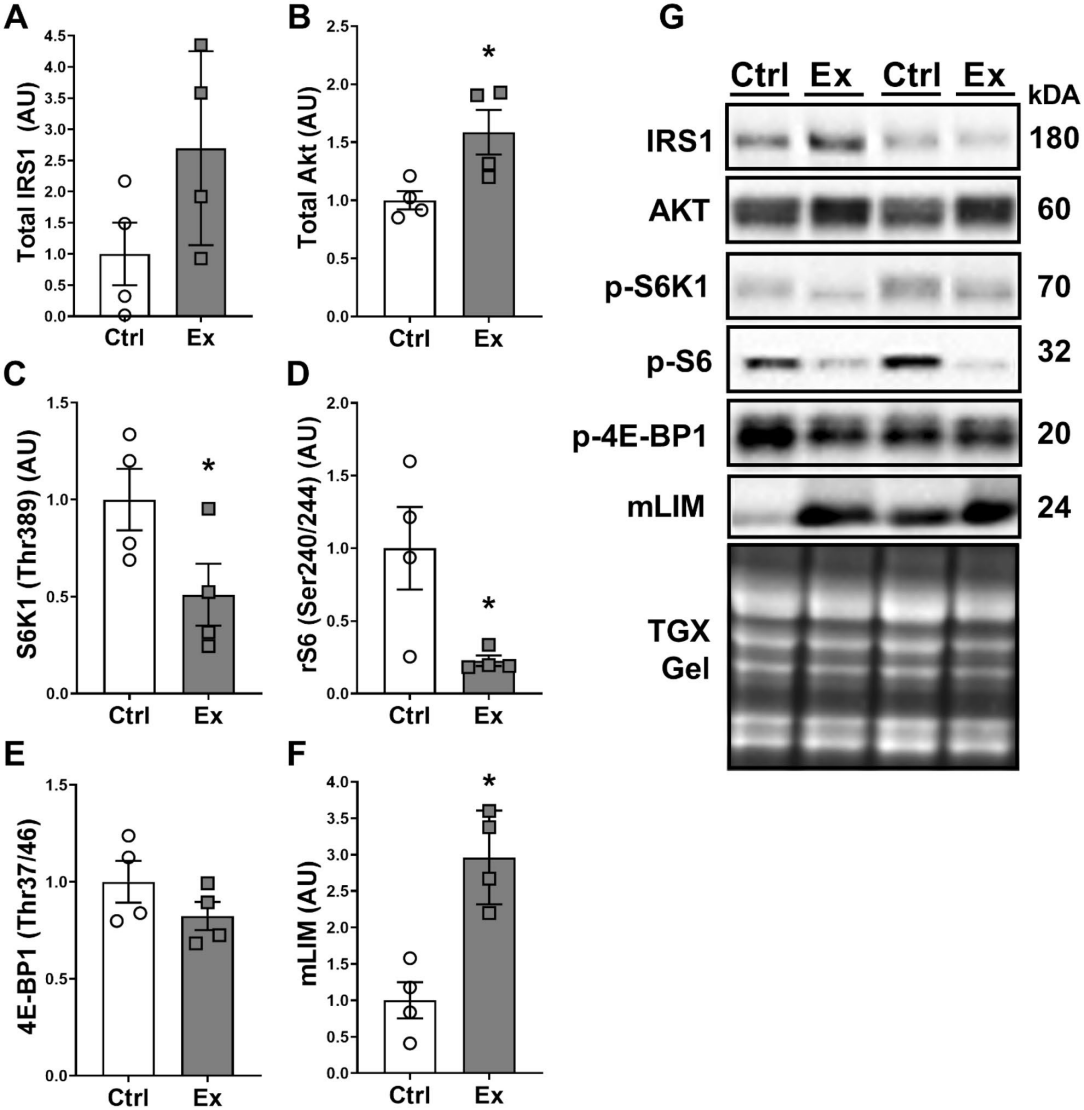
Baseline anabolic signaling in response to chronic exposure to eccentric contractions. Protein levels of total IRS1 (**A**), total Akt (**B**), phosphorylated S6K1 (Thr389) (**C**), phosphorylated S6 (Ser240/244) (**D**), phosphorylated 4E-BP1 (**E**) and mLIM (**F**). Representative blots of all proteins and total protein content of the gel are displayed in (**G**). Pictures of the membranes were cropped to display proteins and samples of interest. Protein levels in (**A–F**) are displayed relative to the non-exercised contralateral control leg. Animals were stimulated four times over two weeks, collection time point was 48 h after the last exercise bout with all animals being in a fasted state (n = 4). Intensity of the bands was normalized to total protein content of the gel or membrane. Data is represented as mean ± SD * indicates a p-value of < 0.05.

In the chronic experiment, 48 h after the last bout many signals that were elevated after an acute bout of exercise were downregulated in the repeatedly stimulated leg. Phosphorylated S6K1 (Thr389) decreased 50% in the stimulated compared to the control leg (p < 0.05) (Fig. 2C). Similarly, its downstream target phosphorylated S6 (Ser240/244) was decreased ~ 75% in the stimulated compared to the control leg (p < 0.05) (Fig. 2D). Additionally, there was a trend for a decrease in phosphorylated 4E-BP1 (Thr37/46) in the stimulated leg (p = 0.1) (Fig. 2E). Simultaneously, upstream of growth factor activation of mTORC1, IRS1 levels tended to increase to 2.7-fold in the stimulated leg compared to the control leg (p = 0.06) (Fig. 2A). Similarly, total Akt levels were increased by 59% in the stimulated compared to the control leg (p < 0.05) (Fig. 2B). Finally, mLIM protein levels were increased to about threefold in the stimulated compared to the control leg (p < 0.01) at rest (Fig. 2F). Representative pictures of the blots are displayed in Fig. 2G.

### No changes to myofiber size, muscle mass or body weight following chronic exercise

**Figure 3:**
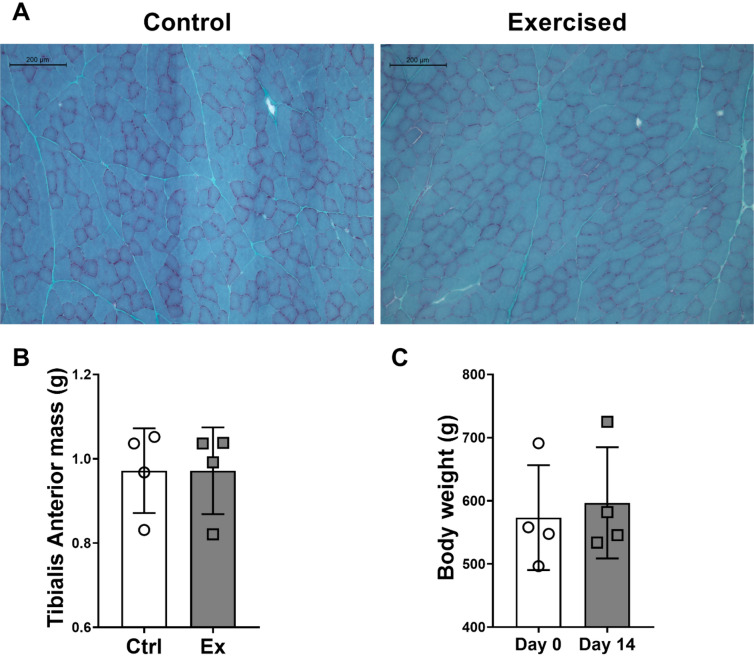
Muscle cross sections and body characteristics after chronic exposure to eccentric contractions. Gomori trichrome stainings show no obvious change in respect to myofiber size or number, and no change in pathophysiological characteristics such as central nuclei, remodeling fibers, connective tissue infiltration or denervation (**A**). No change in muscle mass was observed over the 2-week time frame (**B**). Body weight of the animals remained unchanged over the course of the intervention (**C**). Data is represented as mean ± SD.

We performed histological assessment of the TA using a standard Gomori trichrome staining. We found no obvious changes in myofiber size, central nuclei, connective tissue accumulation, denervation, or regenerating fibers with chronic exercise (Fig. 3A). In line with this, mass of the TA was unchanged between the control leg (0.97 ± 0.1 g) and the exercised leg (0.97 ± 0.1 g) (p = 0.99) (Fig. 3B). We limited food access throughout the intervention to keep the body weight of the animals stable; as a result, no significant increase in body weight was observed (Fig. 3C).

### Chronic eccentric contractions increase myofibrillar protein synthesis

**Figure 4:**
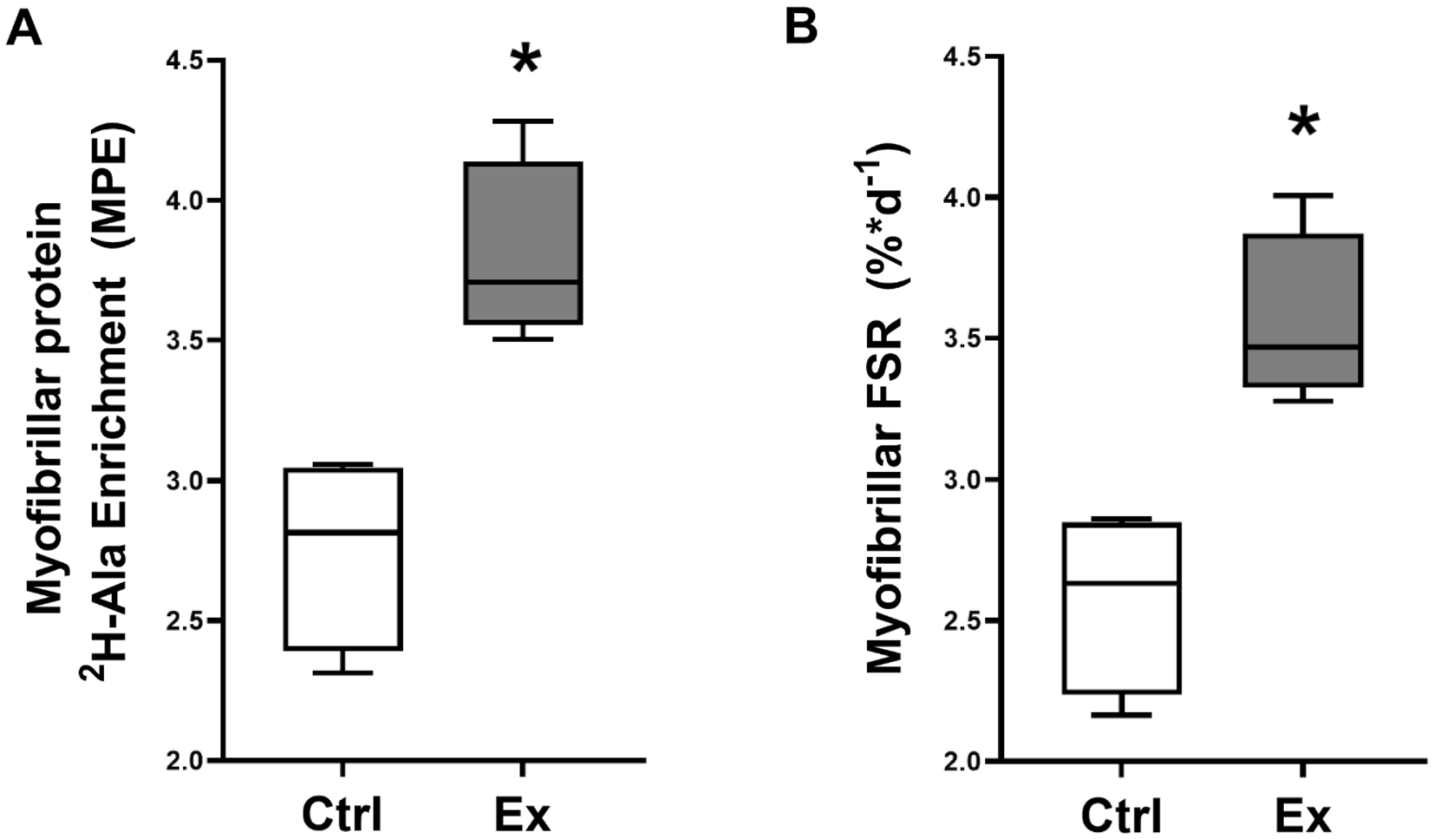
Changes to myofibrillar protein turnover with chronic exposure to eccentric contractions. Incorporation of deuterium-labelled alanine (d-ala) into the myofibrillar muscle fraction of TA was assessed via GC–MS (n = 4). Enrichment of d-ala was increased in the chronically exercised muscle compared to the contralateral control leg (**A**). Similarly, fractional synthetic rate (FSR) of d-ala between circulation and the myofibrillar subfraction of the TA was significantly increased with chronic eccentric contractions compared to the contralateral control condition (**B**). * indicates a p-value of < 0.05. The floating boxes represent the data between the 25th and 75th percentile of the results, the line is the median and the whiskers are the minimum and maximal values.

We measured the incorporation of deuterium labelled alanine from the circulation into the myofibrillar fraction of the TA over the course of two weeks. The enrichment of ^2^H-ala label in the protein bound myofibrillar fraction of the TA increased from 2.8 (± 0.4) mole percent excess (MPE) in the control leg to 3.8 (± 0.3) MPE in the chronically exercised leg (p < 0.05) (Fig. 4A). Similarly, myofibrillar protein synthesis rates in the control leg were 2.6% per day (± 0.3%) but about 38% higher in the chronically exercised leg (3.3 ± 0.3% per day) (Fig. 4B) in good agreement with the acute change in protein synthesis measured by SuNSET (Fig. 1).

## Discussion

This study addressed the complex relationship between molecular signaling, muscle protein synthesis and morphological adaptations by examining changes from an acute bout of eccentric contractions to a second bout and finally an early chronic setting of repeated bouts over multiple weeks. We found that an acute resistance-type exercise stimulus is associated with a robust increase in anabolic signaling and protein synthesis in the hours after exercise; however, this signal is significantly dampened after a second bout. The decrease in molecular signaling with repeated stimuli became even more pronounced at rest after two weeks of eccentric contractions, decreasing below control for p-S6K1 (Thr389) and p-rpS6 (Ser240/244). Interestingly, despite the decrease in baseline anabolic signaling, myofibrillar protein synthesis was substantially higher in the stimulated leg. Despite the increase in myofibrillar protein synthesis, a training induced increase in myofiber size or muscle mass was not observed after two weeks of training. These data highlight the non-linear relationship between acute anabolic signaling, muscle protein turnover and the long-term adaption of muscle hypertrophy.

Classically, the response to resistance type exercise is characterized as an increase in anabolic signaling that results in upregulated synthesis of contractile proteins and eventually muscle fiber hypertrophy^3^. However, even though these experiments identified an association between the acute activation of the mTORC1 signaling pathway and long-term muscle growth, the extent to which there was a causal relationship between the acute signal (i.e., from a first exercise bout) and chronic adaptations is unclear. The strength of this study was to fill in some of the gaps in our understanding between the acute molecular signaling following a first (untrained) exercise bout, that was immediately followed by an analysis of the molecular signature of a second bout, before investigating chronic changes to protein turnover. Previous studies have commonly directly compared acute and chronic adaptations to exercise, without investigating how rapidly after a first bout these changes might already occur.

A first bout of resistance type exercise, and specifically eccentric contractions, is known to cause substantial muscle damage that results in delayed onset muscle soreness, inflammation and swelling of the tissue^24,25^. This extreme response becomes less pronounced with every following stimulus and the tissue appears largely protected from excessive damage once acclimated to the training. This process of rapid adaptation is called the repeated bout effect^26^. Therefore, it stands to reason that part of the molecular signature of a first exercise bout might not be due to mechanotransduction signals that causally relate to contractile protein accrual, but rather the response to acute muscle damage.

Recent data supports this idea of an amplified “anabolic response” due to muscle damage. Mitchell and colleagues found that acute rates of myofibrillar protein synthesis are a poor predictor of long-term muscle growth with resistance type exercise^11^. The same group later found that while protein synthesis does not correlate with long-term growth after a first bout, the change in protein synthesis rates following acclimatization to the resistance type exercise program correlate well with muscle growth at the end of the program^13^. Indeed, they found that the early amplification of myofibrillar protein synthesis was directly associated with structural muscle damage, whereas the correlation between protein synthesis and growth after acclimatization was unrelated to muscle damage. This suggests that mTORC1 activity and increased muscle protein synthesis rates after a first bout largely reflect an adaptive response to exercise and muscle remodeling, rather than muscle growth per se.

In both kinase-dependent and independent manners, mTORC1 is required for muscle repair following injury^27^, suggesting that the acute mTORC1 time course response could be biased by the inflammatory response, or other damage response mechanisms, to unaccustomed exercise. Consistent with this hypothesis, Trappe et al. demonstrated that anti-inflammatory drugs reduce protein synthesis in human skeletal muscle after acute eccentric exercise^28^, possibly by blocking the activity of cyclooxygenase enzymes which stimulate mTORC1^29^ and muscle cell growth^30^, and regulate muscle regeneration^31^. Broadening this hypothesis, recent studies found that canonical anabolic signaling pathways and myofibrillar protein synthesis can be increased in situations of severe muscle damage or atrophy, where a concomitant increase in proteolysis outpaces anabolism resulting in atrophy^32–37^.

Our finding that chronic exposure to resistance type exercise results in a decrease in anabolic signaling is supported by a clinical trial from Phillips and colleagues, in which the authors observed a genetic signature consistent with mTORC1 inhibition that was correlated with gains in muscle mass over 20 weeks of resistance type exercise^10^. This is in line with data on the protein level from Ogasawara et al. in rats, who found that 12 bouts of resistance type exercise led to a decrease in S6K1 activity which could be reestablished through a 12-day break before the 13th bout^5,38^. Similar to our second experiment, Glynn et al. found that physically active rats had reduced baseline S6K1 (Thr389) levels compared to sedentary controls^39^. Multiple clinical trials in humans found that in chronically trained muscle, anabolic signaling through mTORC1 after exercise becomes less pronounced^40–44^ while MPS still increases, albeit in a dampened or shortened manner^45–48^. In our animal model, we found a robust increase in integrated MPS over the two-week course of stimulation. A possible explanation for why MPS is relatively robustly increased in our chronic rat model compared to the clinical trial data in chronically exercised humans is that we measured MPS over the entire two-week intervention period. In other words, MPS in our experiment is an average of the acute response to exercise, likely including a damage-induced amplification of MPS, as well as the response to later stimulation bouts which is likely to be attenuated. An additional explanation along the same line is that while the acute response of MPS to exercise is usually decreased in the trained state, basal MPS has been found to be increased^49^. Therefore, since our measurement of MPS captured exercise induced as well as basal rates, it is possible that increases in the latter contributed to the overall increase observed in our study.

Interestingly, studies over the last decade have also shown that aging causes increased baseline mTORC1 activity in stem cells^50^, liver^51^ and muscle^6,7^, and that inhibition of mTORC1 through rapamycin is able to reduce age-associated muscle loss and extend lifespan in model organisms^6–8,52^. Phosphorylation of the downstream targets of mTORC1, S6K1 and S6, is linked to a negative feedback loop that decreases IRS1 levels and insulin sensitivity^53^. This has caused the hypothesis that age-induced hyperactivity of mTORC1 plays a key role in the development of insulin resistance and diabetes, supported by data that showed that the absence of S6K1 improves insulin sensitivity and glucose homeostasis^54^. To this end, decreased insulin sensitivity in skeletal muscle is the main problem underlying type 2 diabetes^55^ which in turn is closely associated with anabolic resistance and sarcopenia^56,57^. In line with this, we have recently confirmed that the age-induced increase in mTORC1 activity negatively feeds back on IRS1 levels resulting in decreased IRS1 protein levels and a shortened muscle protein synthesis response to resistance type exercise^14^. In humans, immediately after a single bout of resistance exercise insulin sensitivity decreases^58^, whereas following training insulin sensitivity increases concomitant with an increase in IRS1 levels^59^. In the current study, chronic stimulation decreased baseline S6K activity, IRS1 levels tended to increase along with total Akt and myofibrillar protein synthesis in a muscle-specific manner (i.e. only in the exercised muscle). The increase in Akt could be important in the increase of MPS since blocking Akt phosphorylation is necessary to completely blunt the protein synthetic response to resistance exercise and significantly decreases basal skeletal muscle protein synthesis^60^.

A limitation of this study is the relatively short duration of the chronic training stimulus. It is possible that 14 days were insufficient to cause significant changes in myofiber size or muscle mass. However, it is important to keep in mind that by directly stimulating the sciatic nerve in our animal model, we are causing a contraction intensity that is beyond what could be achieved in wake men or rats. This, in addition to the already faster metabolism of rodents compared to men^61^, causes a more intense stimulus that is followed by an accelerated time course of adaptation. Previous studies by our laboratory found that 6-weeks of the same stimulation protocol led to a ~ 14% increase in muscle mass^3^. The more extreme method of synergist ablation causes increases of over 50% in muscle mass of rodents in just 9 days. Even in humans, a recent study has found that as little as four weeks of resistance exercise are sufficient to detect changes in muscle mass^62^. Therefore, we believe that the time frame was sufficient to detect at least tendencies in our animal model. The fact that we did not see such tendencies indicates that the adaptive response to eccentric exercise in respect to the molecular signature as well as MPS are largely independent and timely dissociated from phenotypical changes to size. Another potential reason for the absence of muscle growth in our model could be that we restricted energy intake to keep our animals relatively weight stable. The absence of a calorie surplus that usually occurs in laboratory animals on an ad libitum diet^63^, may have decreased their ability to add de novo muscle tissue to their body. However, the role of total energy availability for skeletal muscle growth in response to resistance exercise is not very well understood and a benefit for increased energy intake has yet to be supported by direct evidence^64^.

Taken together, the collective reports in the literature and the data from this current study suggest that to maintain muscle mass with aging and for muscle to adapt to exercise stimuli, perpetual mTORC1 activity is undesirable. Instead, high degrees of activation of mTORC1 in the early phase following exercise facilitate acute increases in protein synthesis and muscle remodeling, which is followed by the downregulation of mTORC1 signaling components in a more chronic setting. This decrease in mTORC1 is accompanied by a concomitant upregulation of IRS1/Akt that leads to greater insulin sensitivity and nutrient sensing, resulting in elevated muscle protein turnover rates in exercise-adapted muscle.

## Supplementary Information

Supplementary Information.

## Abbreviations

MPS: Muscle protein synthesis
D2O: Deuterium oxide
MPE: Mole percent excess
FSR: Fractional synthetic rate
GC–MS: Gas chromatography–mass spectrometry
mTORC1: Mammalian target of rapamycin complex 1
TA: Tibialis anterior
EDTA: Ethylenediaminetetraacetic acid
EGTA: Ethylene glycol tetraacetic acid
DC: Detergent compatible
PVDF: Polyvinylidene difluoride
TBST: Tris-buffered saline with 0.1% Tween-20
SLB: Sucrose lysis buffer
MTBSTFA: *N*-*tert*-Butyldimethylsilyl-*N*-methyltrifluoroacetamide
TBDMS: *tert*-Butyldimethylsilyl
