# Supplementary material for: Myofibrillar protein synthesis rates are increased in chronically exercised skeletal muscle despite decreased anabolic signaling
Source: Sci Rep. 2022 May 9;12:7553. doi: 10.1038/s41598-022-11621-x (PMC9085756; doi:10.1038/s41598-022-11621-x)

Gel1, Membrane 3

Sample order: Control, Stimulated, Control, Stimulated, Control, Stimulated, Stimulated, Control, Protein Ladder

Total IRS1 (Figure 2A in the manuscript)


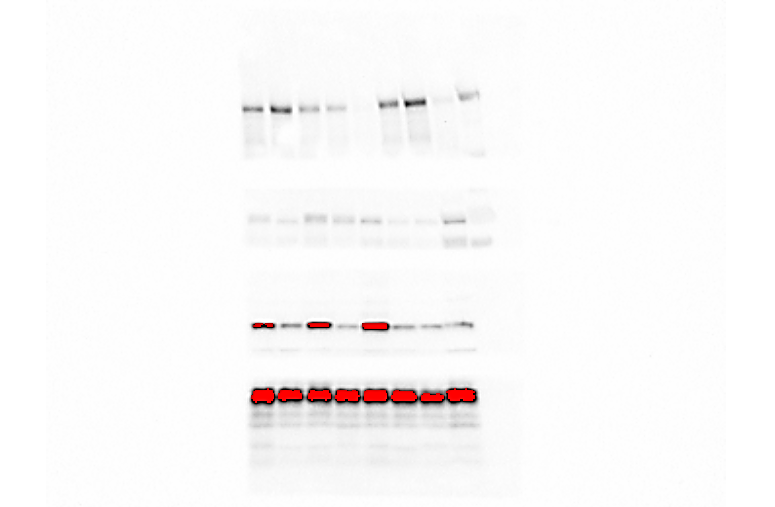


S6K (Thr389) (Figure 2C in the manuscript)


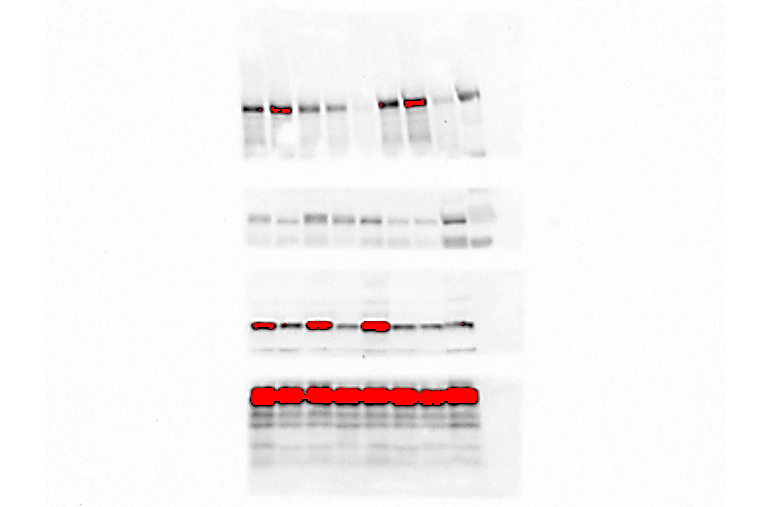


rpS6 (Ser240/244) (Figure 2D in the manuscript)


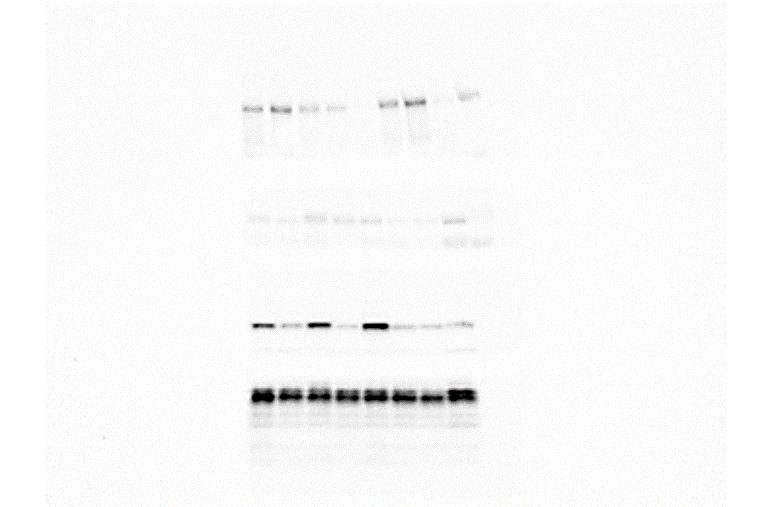


4E-BP1 (Thr37/46) (Figure 2E in the manuscript)


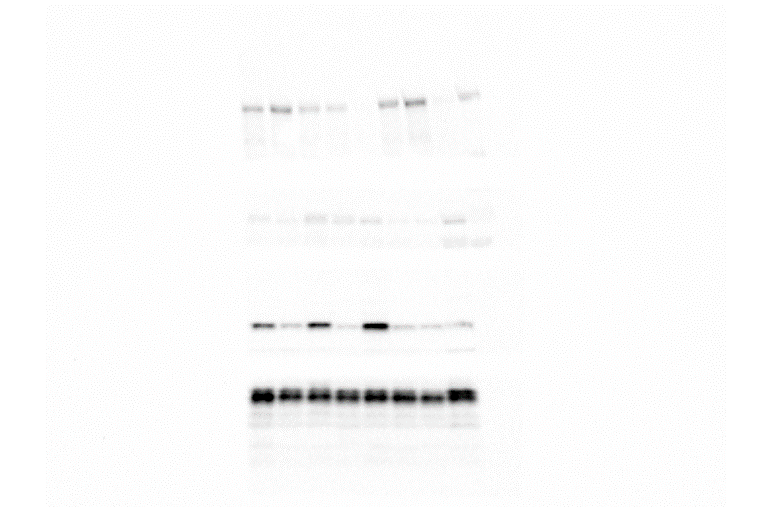


Gel1 (membrane 3)


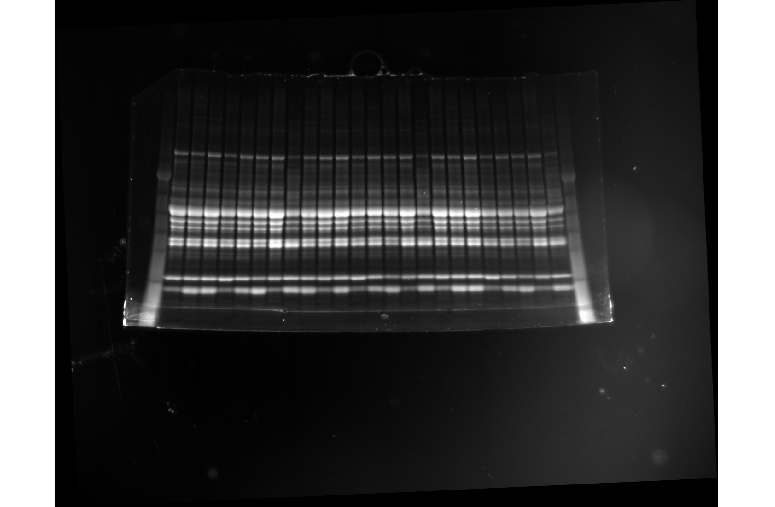


Gel1, membrane1

Sample order: Control, Stimulated, Control, Stimulated, Stimulated, Control, Control Stimulated

mLIM (CSRP3) (Figure 2F in the manuscript)


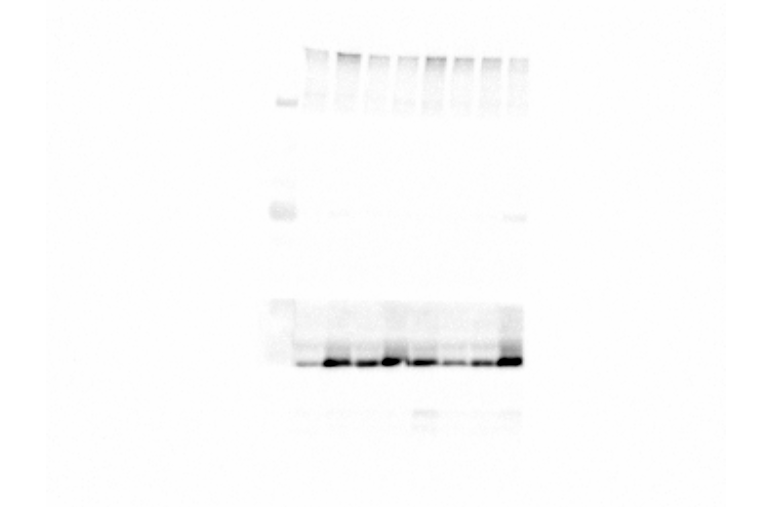


Gel 1 (membrane 1)


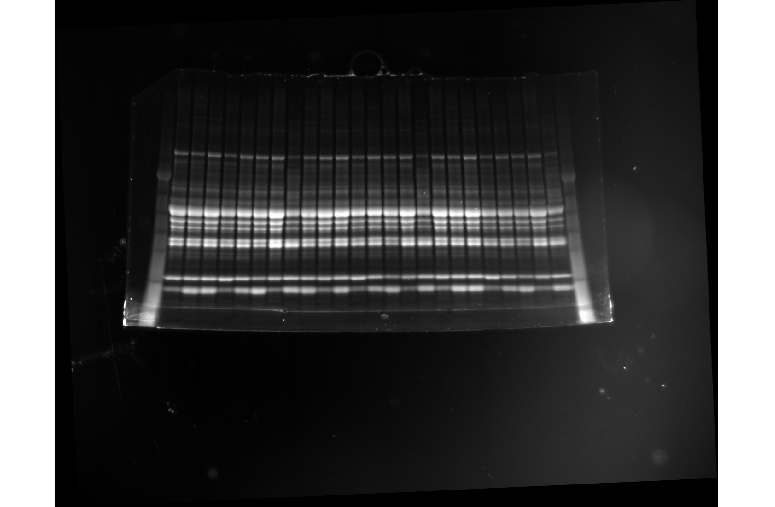


Gel 2, membrane 1

Sample order: Control, Stimulated, Control, Stimulated, Stimulated, Control, Control Stimulated

Total Akt (Figure 2B in the manuscript)


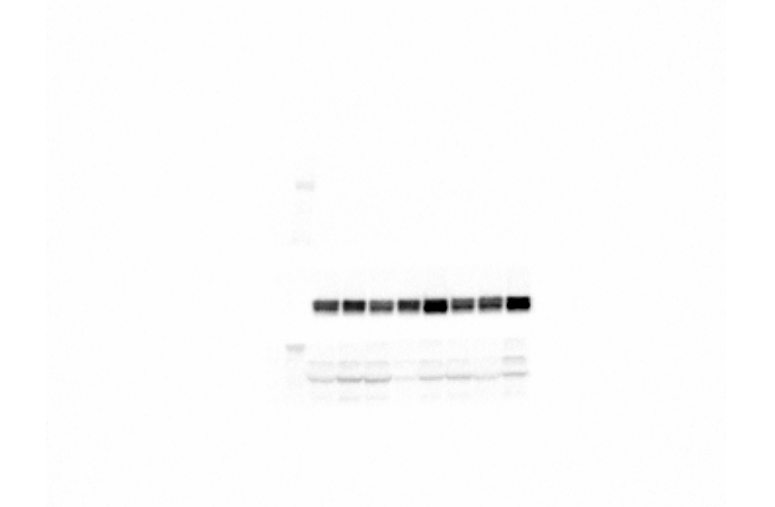


Gel 2 (membrane 1)


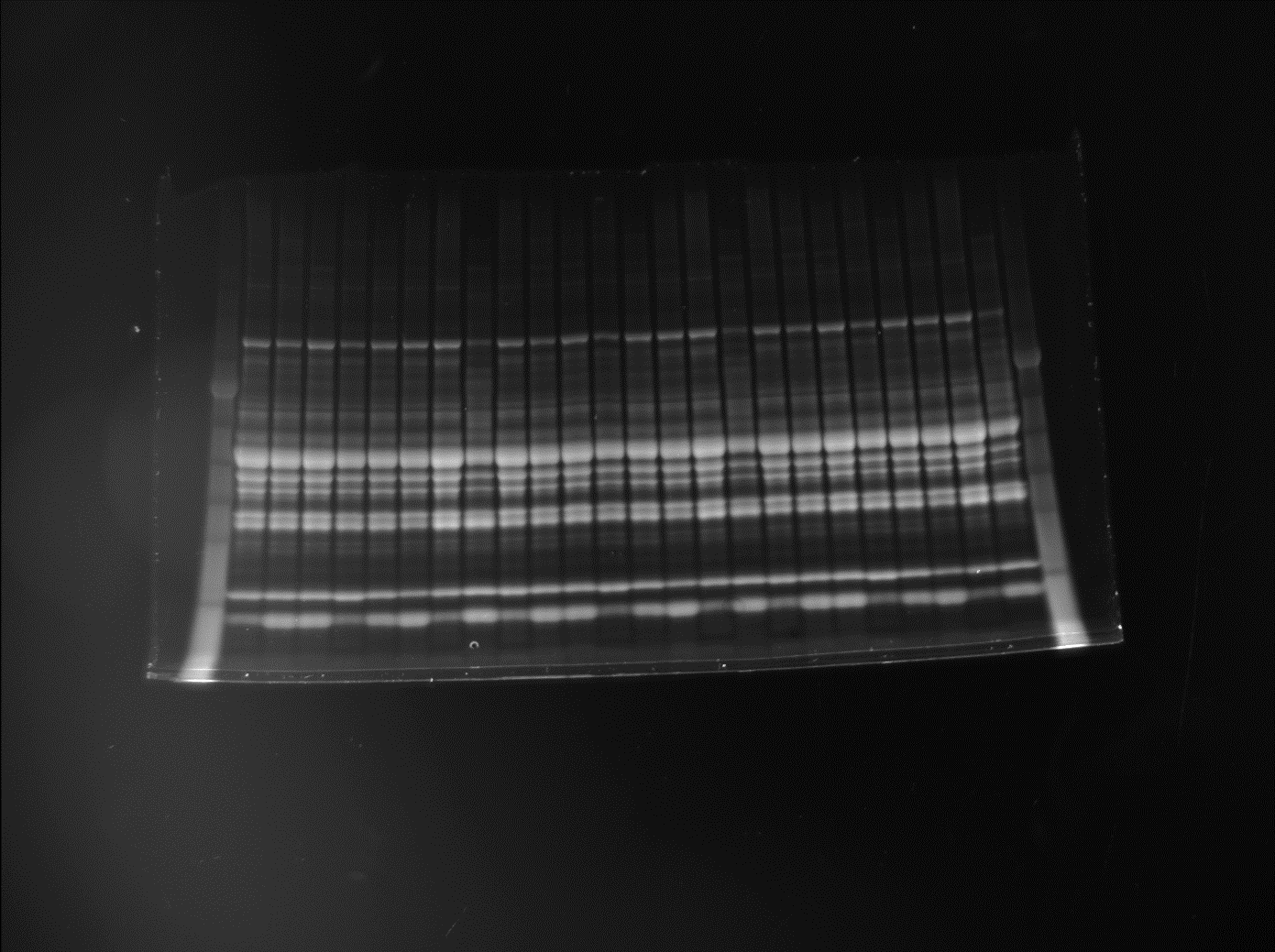

Supplement: Supplementary file 1 — Supplementary Information. [file 41598_2022_11621_MOESM1_ESM.docx]
